# Pulmonary salivary gland tumor–hyalinizing clear cell carcinoma: a literature review

**DOI:** 10.1186/s13000-024-01460-x

**Published:** 2024-02-22

**Authors:** Xinyuan Wang, Shumin Hu, Hongyang Lu

**Affiliations:** 1 Zhejiang Key Laboratory of Diagnosis & Treatment Technology On Thoracic Oncology (Lung and Esophagus), Zhejiang Cancer Hospital, Hangzhou, 310022 Zhejiang China; 2 Department of Thoracic Medical Oncology, Zhejiang Cancer Hospital, Hangzhou, 310022 Zhejiang China; 3 Hangzhou Institute of Medicine (HIM), Chinese Academy of Sciences, Hangzhou, 310018 Zhejiang China; 4 Postgraduate Training Base Alliance of Wenzhou Medical University, Wenzhou, 325035 People’s Republic of China; 5 The Second Clinical Medical College, Zhejiang Chinese Medical University, Hangzhou, 310053 People’s Republic of China

**Keywords:** Pulmonary HCCC, Pathology, Molecular characteristics, *EWSR::ATF1*

## Abstract

Primary pulmonary hyalinizing clear cell carcinoma (HCCC) is a very rare lung tumor that accounts for less than 0.09% of all primary lung tumors and has no specific epidemiology. The correct diagnosis requires imaging, laboratory, pathological, immunohistochemical, and molecular examination. The most typical feature of pulmonary HCCC is the clear cell component with clear stroma. In addition, the fusion gene *EWSR1::ATF1* due to t(12;22)(q13;q12) is essential for the pathological diagnosis of pulmonary HCCC. The main treatment for pulmonary HCCC is surgery. This review focus on the pathological features, immunohistochemical examination, mutation analysis and treatment of pulmonary HCCC.

## Introduction

Hyalinizing clear cell carcinoma (HCCC), also referred to as clear cell carcinoma (CCC), is a rare salivary gland tumor usually caused by the salivary glands of the head and neck [1]. Salivary gland-type tumor (SGT) presenting outside the head and neck is a rare phenomenon, with pulmonary SGT being less than 1% of all lung tumors [2, 3]. Primary pulmonary hyalinizing clear cell carcinoma, a type of pulmonary SGT, accounts for less than 0.09% of all primary lung tumors and was first reported in 2015 [4, 5]. Pulmonary HCCC is a low-grade malignant tumor [6] with histological, immunophenotypic and molecular features similar to those of salivary gland CCC of the head and neck [7]. The epidemiological and clinical features of pulmonary HCCC are neither specific nor representative [5]. Therefore, the correct diagnosis of pulmonary HCCC requires a combination of histopathological examination, immunohistochemistry (IHC) and molecular mutation analysis. Pathologically, there are several distinctive features such as a clear cell components, hyalinizing stroma, and low-grade cytological atypia [5]. In terms of molecular fusion, Ewing’s sarcoma break region 1 (*EWSR1*) rearrangement was detected in 87–91% of HCCCs [8]. The most common genetic variant is t(12;22)(q13;q12), leading to the gene fusion *EWSR1* activation of transcription factor 1 (*ATF1*) [9, 10]. Other gene fusions, such as *EWSR1::CREM* and *ATF1::SPLTC2*, have been reported in pulmonary HCCC [6]. To date, surgery is recognized as the main treatment of pulmonary HCCC [7], while the effectiveness of chemotherapy and radiotherapy is unclear [11].

## Methods

We used a comprehensive computer search of literature in the National Institutes of Health PubMed database, using combinations of the terms pulmonary hyalinizing clear cell carcinoma, clear cell carcinoma, clear cell and salivary gland tumor to search for studies published from 2015 to December 2023. After a careful review of each published case, tumors were selected for inclusion if they were diagnosed as pulmonary HCCC. Then, extract data related to demographics, clinical presentation, radiological presentation, gene fusion, treatment and outcomes.

### Clinical characteristics

HCCC usually originates from the salivary glands of the head and neck and is a common low-grade malignant tumor of the salivary gland [1, 4]. Uncommon locations include trachea, bronchi, and nasopharynx [12]. Pulmonary HCCC accounts for less than 0.09% of primary lung tumors [5]. Our review of the previous literature showed that the average age of pulmonary HCCC patients is 54 years old, and tumors seem to be more common in females, accounting for 61.8% of cases. As is well known, lung cancer is closely related to smoking; however, 76.5% of pulmonary HCCC patients have no history of smoking. The detailed information of pulmonary HCCC cases is shown in Table 1. Pulmonary HCCC originates in the submucosal bronchial glands and presents as a central lesion of an intrabronchial mass, so patients usually present with signs and symptoms of bronchial obstruction, including shortness of breath, cough, chest pain, hemoptysis, pneumonia or fever [11]. Sometimes, patients are asymptomatic and can only be detected by physical examination [13].

**Table 1:** Summary of clinicopathologic features of collected pulmonary hyalinizing clear cell carcinoma

Ref	Years, Sex and smoker history	Size and Location	Molecular Testing		Treatment	Lymph node status				Follow-up Time/ Outcome
[1]	66F; Ex-smoker	1.3 cm; trachea		EWSR1 gene rearrangement	Laser therapy + cryotherapy			NM	NM	
[4]	38 M; Non-smoker	2.6 cm; trachea	EWSR1 gene rearrangement EWSR1::ATF1 gene fusion		Lobectomy			NM	No recurrence or metastasis after 10 mo	
[5]	56F; Non-smoker	3.3 cm; right segmental bronchus	EWSR1 gene rearrangement EWSR1::ATF1 gene fusion		Lobectomy			( +)	No recurrence or metastasis after 12 mo	
[5]	35F; Smoker	2.8 cm; right segmental bronchus	EWSR1 gene rearrangement EWSR1::ATF1 gene fusion		Lobectomy			(-)	No recurrence or metastasis after 79 mo	
[5]	52F; Non-smoker	3.3 cm; right segmental bronchus	EWSR1 gene rearrangement EWSR1::ATF1 gene fusion		Lobectomy			NM	No recurrence or metastasis after 181 mo	
[6]	Sex F: M = 5:3 Average age: 58 years Non-smoker: smoker = 7:1	Average size:2.1 cm right lung: left lung: trachea = 4:3:1	8 cases EWSR1 gene rearrangement 5 cases EWSR1::ATF1 fusion gene (one with an ATF1::SPTLC2 gene) 1 cases EWSR1::CREM fusion gene 2 cases no mention		Lobectomy			1/8( +) 7/8(-)	1 case was lost, 7 patients were followed up for 6 to 45 months, all without recurrence	
[7]	54F; ex-smoker	3.5 cm; Left upper lung lobe	EWSR1 gene rearrangement EWSR1::ATF1 gene fusion		lobectomy			(-)	No recurrence or metastasis after 16 mo	
[12]	44 M; Non-smoker	3.5 cm;lobar bronchus (left lower)	EWSR1 gene rearrangement		Lobectomy			NM	Recurrence-free 4 years after surgery	
[12]	56F;Non-smoker	1.6 cm;lobar bronchus (left upper)	EWSR1 gene rearrangement		Lobectomy			NM	NM	
[12]	44F;Non-smoker	1.3 cm; lobar bronchus (right upper)	EWSR1 gene rearrangement		Lobectomy			NM	NM	
[12]	33F;Non-smoker	4.9 cm; lobar bronchus (right middle)	EWSR1 gene rearrangement		Lobectomy			NM	NM	
[12]	64 M;Non-smoker	4.9 cm;left upper lobe hilm	EWSR1 gene rearrangement		Lobectomy + chemotherapy			( +)	Recurrence free 9 month after surgery	
[13]	57F;Ex-smoker	2.8 cm; right lower lobe	EWSR1 gene rearrangement; EWSR1::ATF1 gene fusion		Lobectomy			( +)	No recurrence or metastasis after 3mo	
[14]	53 M;Non-smoker	1.6 cm; bronchus	EWSR1 gene rearrangement; EWSR1::ATF1 gene fusion		Lobectomy			( +)	Metastasis after 192 mo	
[15]	32 M;Non-smoker	1.8 cm; left lower lobe	EWSR1 gene rearrangement		Lobectomy			(-)	No recurrence or metastasis after 18 mo	
[15]	39 M; Non-smoker	2.6 cm;right lower lobe	EWSR1 gene rearrangement		Lobectomy			(-)	No recurrence or metastasis after 18 mo	
[16]	55 M;Ex-smoker	2.5 cm; bronchus	EWSR1 gene rearrangement		Lobectomy			(-)	No recurrence or metastasis after 20 mo	
[17]	58 M;Non-smoker	4.3 cm; right upper lobe	EWSR1 gene rearrangement; EWSR1::ATF1 gene fusion		lobectomy + Chemotherapy			NM	No recurrence or metastasis after 10 mo	
[17]	60F;Non-smoker	2 cm; left lower lobe	EWSR1 gene rearrangement; EWSR1::ATF1 gene fusion		lobectomy + lymphadenectomy			(-)	No recurrence or metastasis after 10 mo	
[18]	55F;smoker	2.5 cm; trachea	EWSR1 gene rearrangement		Resection + radiation therapy			(-)	No recurrence or metastasis after 12 mo	
[19]	46F;Non-smoker	Size no mention; trachea	EWSR1 gene rearrangement; EWSR1::ATF1 gene fusion		Resection + chemoradiation			(-)	Deceased 6 years after first diagnosis	
[20]	64F;Non-smoker	4.2 cm; left upper lobe of lung	EWSR1::CREM gene fusion		Lobectomy + lymph node dissection			(-)	No recurrence or metastasis after 2 mo	
[21]	66 M;Ex-smoker	3.3 cm; right main bronchus	EWSR1 gene rearrangement; EWSR1::ATF1 gene fusion		Resection			NM	Recurrence after 2 years	
[21]	48F;Non-smoker	2 cm; right middle lobe	EWSR1 gene rearrangement; EWSR1::ATF1 gene fusion		Double lobectomy of right middle and lower lobe			NM	No recurrence or metastasis after 14 mo	
[22]	75F; Smoker history no mention	0.9 cm; lower lobe of the lung	FISH: EWSR1 gene rearrangement; EWSR1::CREM gene fusion		Lobectomy			(-)	No evidence of disease at 8 mo postsurgery	
[23]	81 M;Non-smoker	2.9 cm; right upper lung	EWSR1::ATF1 gene fusion		Lobectomy + lymph node dissection			( +)	No recurrence or metastasis after 9 mo	
[24]	54F;Non-smoker	2.5 cm; right Upper Lung Lobe	EWSR1 gene rearrangement; EWSR1::ATF1 gene fusion		Lobectomy + regional lymph node dissection			(-)	No recurrence or metastasis after 8 mo	
Summary	F:M = 21:13	Average size:2.6 cm	FISH: 33 cases EWSR1 gene rearrangement EWSR1::ATF1:EWSR1::CREM = 19:3		Lobectomy(main)		Rarely suffer		Positive prognosis	

*F* Female, *M* man, *NM* Not mention, *FISH* Fluorescence in situ hybridization, *RT-PCR* Reverse transcription-polymerase chain reaction, *mo* Month

The Ki-67 index of tumor cells in the pulmonary HCCC is low, with a positive index of 1% ~ 15%, indicating a slow growth rate [5, 6]. The mitotic rate is usually low, and necrosis is rare [25], indicating that this tumor is of low grade. In the head and neck, HCCC commonly shows inert clinical behavior, local recurrence and locoregional nodal metastasis of 49% and 15%, respectively, have been reported [26]. Although pulmonary HCCC is similar to salivary gland HCCC and has a lower likelihood of metastasis, local recurrence may still occur [1, 4]. In some cases of pulmonary HCCC, tumor cells infiltrating the lung parenchyma [14], cartilage [4, 15, 16], and nerves [5, 16, 17] have been confirmed, which may indicate an aggressive clinical course that can also lead to metastasis and recurrence [14]. For example, Wang et al. [14] diagnosed a patient with pulmonary HCCC who underwent right upper lobe lobectomy. 16 years later, four recurrent, 1.5 cm diameter hard white pulmonary HCCC tumors were found in the right lower lobe, and enlarged group 3/4 lymph node metastases were observed.

Taken together, pulmonary HCCC is a rare tumor that occurs unrelated to smoking and originates from submucosal bronchial glands. In addition, due to the lack of relevant reports and the long duration of metastasis and recurrence, little is known about pulmonary HCCC. Therefore, we need more case studies to illuminate the clinical characteristics of pulmonary HCCC.

### Imaging examination

Chest imaging is the preferred method to detect pulmonary HCCC, especially computed tomography (CT) scan is advantageous as it can clearly display the lesion site, size, morphology, margin, density, presence of pleura, pericardial invasion, and enlargement of mediastinal lymph nodes in the hilum. However, the imaging features of pulmonary SGT were not distinct. CT shows a well-defined single mass or nodule in the bronchus, causing bronchial wall thickening and/or intramural polyp-like growth [11]. On positron emission tomography—computed tomography (PET-CT), this tumor shows FDG avidity like squamous cell carcinoma [27]. All pulmonary HCCC masses are central, involving the trachea, main bronchus, lobar bronchus and segmental bronchial mucosa [6]. Endobronchial lesions are often observed as imaging characteristic of obstructive pneumonia and/or pulmonary atelectasis [11].

### Pathology and immunohistochemistry examinations

The histological, immunophenotypic and molecular characteristics of pulmonary HCCC are similar to those of salivary gland CCC in the head and neck [7]. Primary salivary gland tumors of the tracheobronchial tree originate from the submucosal glands of the bronchi [18]. Pulmonary HCCC is often located near the bronchus or extends into the bronchial lumen [5], obstructing the bronchus and a large amount of mucus is observed in the sample [4]. Grossly, pulmonary HCCC tumors has a tan-white [4, 11], gray-white [11] or brownish [7] cut with a fibrous and firm consistency, well-defined margins [4, 7, 11, 15, 16], and no epithelium [6].

Pathological examination reveals infiltrative pulmonary HCCC neoplasms composed of irregular nests, cords and trabeculae of polygonal tumor cells [15]. The tumor cells are rather monotonous, small to medium sized, and contain oval nuclei with irregular nuclear contours and ample clear cytoplasm [11]. Sometimes, very rare intranuclear cytoplasmic inclusions found in some pulmonary HCCCs [15]. There is no or only very concentrated necrosis in the tumor cells, and mitotic activity is low [11].Focal mucin pools may be evident [11]. The edge of the tumors is often surrounded by a lymphocytic infiltrate [4, 28]. It is worth noting that, despite the name "clear", most cases of pulmonary HCCC are eosinophilic, and the proportion of cells with clear or eosinophilic cytoplasm varies from case to case, with a clear gradual transition, so the diagnosis of pulmonary HCCC was previously based on the growth pattern (cords, nests, trabeculae, etc.) and the interstitial glassy appearance [5, 6]. Moreover, most pulmonary HCCC tumor cells have clear boundaries, however, under a microscope, it can be observed that pulmonary HCCC tumor cells invade adjacent alveolar parenchyma and lymph nodes around the bronchi or damages bronchial cartilage. In addition to the above situation, tumor cells were seen spreading along the bronchial wall below the respiratory epithelium in the case demonstrated by Feng et al. [17]. Given the rarity of pulmonary HCCC, if these morphological details do not provide a definitive diagnosis, other techniques such as molecular studies or immunohistochemical markers can be applied [11].

The immunohistochemical features of pulmonary HCCC can be summarized by reviewing previous literature. In most cases, p63, p40, CK7, CK5 /6 were positive, while thyroid transcription factor-1 (TTF-1), smooth muscle actin (SMA) and S-100 protein were negative. Immunohistochemical results for patients with pulmonary HCCC are specified in Table 2. P63 was positive, indicating a proliferative potential of the lesion [29]. Since pulmonary HCCC tumor cells were S-100-negative and epithelial membrane antigen-positive, it was suggested that pulmonary HCCC was predominantly epithelial rather than myoepithelial in nature [4]. The absence of focal clear cell features of S-100 and SMA expression in the background of transparent fibrosis indicates that pulmonary HCCC may originate from bronchial submucosal glands [4]. SOX10 was consistently negative in HCCC and mucinous epidermoid carcinoma. Furthermore, SOX10 is a marker of bronchial glandular differentiation, suggesting that pulmonary HCCC are not of acinar cell origin [5]. According to reports, a patient with pulmonary HCCC developed tumor metastases 16 years after right upper lung lobectomy. Interestingly, although the morphological features of this metastatic case were similar to primary pulmonary HCCC, immunohistochemistry was altered and p63 expression was absent in this case [14].

**Table 2:** Immunohistochemical results of pulmonary hyalinizing clear cell carcinoma

Refs	No. of patients	HMB-45	CK20	TTF-1	Napsin A	SMA	S-100	SOX10	p40	p63	CK7	CK5/6
[1]	1	NM	NM	-	-	NM	-	NM	NM	+	+	NM
[4]	1	NM	-	-	-	-	-	NM	+	+	+	NM
[5]	3	3/3-	NM	3/3-	3/3-	NM	NM	3/3-	3/3 +	3/3 +	3/3 +	3/3 +
[6]	8	NM	NM	NM	NM	8/8-	8/8-	4/8 +	5/8 +	5/8 +	8/8 +	NM
[7]	1	-	-	-	-	NM	NM	NM	NM	+	-	+
[12]	5	1/5-	1/5-	4/5-	1/5-	3/5-	5/5-	2/5-	4/5 +	3/5 +	1/5 +	1/5 +
[13]	1	-	-	-	-	-	-	-	+	+	+	+
[14]	1	NM	NM	-	-	-	-	-	-	-	NM	NM
[15]	2	2/2-	2/2-	2/2-	2/2-	2/2-	2/2/-	NM	NM	2/2 +	2/2 +	NM
[16]	1	NM	-	-	-	-	-	NM	+	+	+	+
[17]	2	2/2-	2/2-	2/2-	2/2-	2/2-	2/2-	2/2-	2/2 +	2/2 +	2/2 +	2/2 +
[18]	1	NM	NM	NM	NM	NM	-	-	+	+	+	+
[19]	1	NM	NM	NM	NM	-	-	NM	NM	+	NM	NM
[20]	1	NM	NM	NM	NM	NM	NM	NM	NM	NM	NM	NM
[21]	2	2/2-	NM	2/2-	NM	NM	2/2-	NM	NM	2/2 +	NM	NM
[22]	1	NM	NM	NM	NM	NM	-	-	NM	+	+	NM
[23]	1	NM	NM	NM	NM	-	NM	NM	+	NM	+	+
[24]	1	NM	NM	-	-	-	NM	NM	+	+	+	+
Total	34	(12/16)	(8/12)	(20/21)	(15/19)	(22/24)	(27/27)	(11/22)	(20/24)	(26/31)	(24/28)	(12/16)
Rate	/	75%-	66.7%-	95.2%-	78.9%-	91.7%-	100%-	50%-	83.3%+	83.9%+	85.7% +	75% +

+ : positive, -: nagative, *NM *Not mention, *TTF-1 *Thyroid transcription factor-1, *SMA *Smooth muscle actin

### Molecular characteristics

Fusion genes are important molecular genetic features of many tumors. Translocations is considered to be the first step in tumor development [30], leading to gene fusions that often produce new, tumor-specific chimeric transcription factors [31], which in turn might disrupt gene expression [32]. Many translocations are associated with specific tumor types that have unique clinical features and gene expression profiles [30]. Antonescu et al. [9] identified the *EWSR1* rearrangement in pulmonary HCCC by fluorescence in situ hybridization (FISH), the fusion transcript *EWSR1::ATF1* by reverse transcription-polymerase chain reaction (RT-PCR) [19]. The fusion of *EWSR1::ATF1* in most pulmonary HCCCs is due to t(12; 22)(q13; q12) [9, 10]. Translocation of *EWSR1* is a determinant molecular feature and is present in approximately 80% of salivary CCCs [15]. The *EWSR1::ATF1* fusion is the most common form of fusion in the salivary CCCs and is usually detected in 80% to 90% of cases [6]. *EWSR* and *ATF1* genes are rearranged not only in primary pulmonary HCCC but also in pulmonary HCCC metastases [14]. We reviewed previous literature and found that *EWSR1::ATF1* gene fusion was present in 19/34 cases and *EWSR1::CREM* fusion in 3/34 cases, with one case showing *EWSR1::ATF1* gene fusion and *ATF1::SPLTC2* gene fusion. The detailed gene fusions of the cases are shown in Table 1. Recently, Grosjean et al. [20] reported a case of pulmonary HCCC with a final diagnosis of *EWSR::CREM* fusion after analysis by RNASeq. Diagnosis can be confirmed by demonstrating rearrangements involving *EWSR1::ATF1* or, more rarely, *EWSR1::CREM*. Although, *EWSR1* exhibits the ability to fuse with various partner genes such as *POU5F1*, *PBX1* and *ZNF444,* fusion with *ATF1* at a specific breakpoint (*EWSR1* exon 11:*ATF1* exon 3) was detected only in salivary gland HCCC [9]. In the research by Di et al. [21], fusion of *EWSR1::ATF1* (exon 2: exon 12) was found in a patient, which was the first fusion found in primary lung HCCC except for exon 11 and exon 3, but the significance of fusion is still unclear.

*EWSR1* rearrangements can distinguish pulmonary HCCC from other pulmonary salivary gland tumors. To date, other gene fusions in pulmonary HCCC include *EWSR1::CREM* and *ATF1::SPLTC2*. *EWSR1::ATF1* plays a certain role in the diagnosis of pulmonary HCCC. However, *EWSR1* rearrangements (with different binding partners) are also detected in other tumors, such as hematolymphoid neoplasms, Ewing sarcoma/ primitive neuroectodermal tumors, desmoplastic small round cell tumor, clear cell sarcoma, myxoid chondrosarcoma, myxoid liposarcoma, and melanocytic neoplasms [4]. Although *EWSR1::ATF1* is not specific for the diagnosis of pulmonary HCCC, such as in clear cell sarcomas, angiomatoid fibrous histiocytoma, angiosarcoma, malignant gastrointestinal neuroectodermal tumor, and the soft tissue myoepithelial tumor, *EWSR1::ATF1* can distinguish pulmonary HCCC from other tumors without this gene fusion [4]. In addition, our review of previous literature shows that *EWSR1::CREM* is observed in clear cell sarcomas, clear cell sarcomatoid tumors of the gastrointestinal tract, myxoid fibrous histiocytomas, unclassified spindle cell tumors, and unclassified small round cell tumors [6]. Therefore, the combination of morphological and immunophenotypic features and tumor location deems *EWSR1* rearrangement as a promising indicator for the diagnosis of pulmonary HCCC [8].

### Differential diagnosis

The primary salivary gland type of CCC needs to be distinguished morphologically from solid sheet-like carcinomas such as squamous cell carcinoma of lung, mucinous epidermoid carcinoma, myoepithelial carcinoma, solid subtype adenocarcinoma and other metastatic clear cell carcinomas [6].

Squamous cell carcinoma of lung: pulmonary HCCC can be squamous epithelial metaplasia and needs to be differentiated from squamous cell carcinoma. Squamous cell carcinoma is usually more heterogeneous, with more nuclear schizophrenia and more aggressive growth, and may have *FGFR1*, *DDR2* and *PIK3CA* mutations. The absence of the *EWSR1* fusion gene helps to differentiate it from salivary gland CCC [6].

Mucinous epidermoid carcinoma: pulmonary HCCC may present with mucus secretion and squamous or epidermis-like differentiation and needs to be differentiated from mucinous epidermoid carcinoma. Mucinous epidermoid carcinoma usually presents with distinct mucinous, epidermis-like and intermediate cells, forming solid nests and often cystic changes. Most importantly, mucinous epidermoid carcinoma exist a *MAML2* gene fusion, which helps to differentiate it from pulmonary HCCC [6].

Clear cell myoepithelial carcinoma: the cytoplasm of tumor cells is transparent, mildly atypic, nest-like growth, which is similar to pulmonary HCCC in morphology. *EWSR1* gene transmutation also exists in myoepithelial carcinoma, but the fusion form is different from pulmonary HCCC. *EWSR1::PBX1*, *EWSR1::ZNF444* and *FUS::KLF17* are common [33]. In addition, myoepithelial carcinoma cells are often spindle-shaped, plasma cell-like and epithelioid, and are positive for markers of myoepithelial differentiation (S-100 protein, GFAP, SMA, Calponin, etc.) to varying degrees, whereas in pulmonary HCCC, these markers are negative [6].

Solid subtype adenocarcinoma solid subtype of lung adenocarcinoma cells may appear as clear cells, and it often express TTF-1 and Napsin A, which are markers of lung adenocarcinoma [6] and not of pulmonary HCCC.

Pulmonary metastatic CCC: pulmonary metastatic renal CCCs and ovarian CCCs, all of which may present with clear cells, but positive for *PAX8* in metastatic kidney cancer and ovarian cancer. The differential diagnosis can be made by combining the clinical history, histopathological features and immunophenotype [6].

In addition, since pulmonary HCCC and salivary gland CCC share the same features, consisting mainly of vacuolated cells with large amounts of clear cytoplasm, which might be confused with metastatic salivary CCC. Therefore, thorough clinical examination is essential for excluding metastatic salivary CCC [5, 34]. Finally, PET-CT would clarify the location of the primary site, thus excluding CCC originating from the salivary gland at the primary site.

### Treatment

Due to the low prevalence of pulmonary HCCC, there is no consensus on the best treatment strategy. Nevertheless, it is currently believed that complete surgical resection is the best treatment option [7, 35]. On the aspect of HCCC of the head and neck, Albergotti et al. [36] suggest that for patients with positive margins, re-excision should be attempted to obtain negative margins, and if this is not a feasible option, adjuvant radiotherapy would be appropriate to consider. Gubbiotti et al. [19] had applied radiotherapy and chemotherapy to a patient with pulmonary HCCC, but the efficacy was not very clear and the patient died 6 years after the first consultation. The efficacy of chemotherapy and radiotherapy is unknown because of the small number of cases in which they have been applied [11]. Radiotherapy alone or in combination with radio-sensitization chemotherapy may be useful for recurrent disease, but the evidence is insufficient [37]. Therefore, Radiotherapy and chemotherapy are current directions worth exploring.

Treatment options for pulmonary HCCC include not only surgery, but also laser therapy and cryotherapy. Icard et al. [1] found a 66-year-old woman in respiratory failure due to a pulmonary HCCC mass that blocked 60% of the tracheal lumen. Hence, neodymium-doped: yttrium aluminum garnet (Nd: YAG) laser treatment was performed, and continuous cryotherapy was performed within one to two minute freeze thaw cycles. Cryotherapy to the areas of cauterization offers a localized hemostasis to the damaged mucosa and potentially localized treatment to the tumor. In addition, this local tumor treatment reduces the recurrence rate of the tumor and allows for sloughing of the remaining mucosa, which may help decrease restenosis associated with endothermic damage caused from laser therapy.

Hitherto, the main treatment for pulmonary HCCC is surgery, with cryotherapy and laser treatment also being options, while the effectiveness of radiotherapy and chemotherapy is unknown. Radiotherapy and chemotherapy may be a direction to try when patients are not well treated with surgery. In addition to this, as gene fusions almost always occur in pulmonary HCCC, then targeted therapy is a future direction that could be explored.

### Prognosis

We reviewed previous literature and found that 34 patients were followed up for 2–192 months with only 2 recurrences. Details of the pulmonary HCCC cases can be found in Table 1. Patients with HCCC usually have a relatively good prognosis. A good to excellent prognosis has been reported for head and neck HCCC [7]. The local recurrence rate is 11% to 20%, metastatic disease is rare [9, 28, 36, 38–42]. Of the 111 patients with head and neck HCCC collected by Albergotti et al. [36], 21 had local recurrence. The time to recurrence ranged from 5 to 180 months, with a mean time of 42.1 months. Necrosis, positive cut margins and lymph node status are risk factors associated with recurrence of head and neck HCCC. In particular, lymph node invasion, positive surgical margin status, and tumor cell necrosis are associated with an increased risk of recurrence, with the highest risk of recurrence in the first 2 years and years 5 to 7 [7]. HCCCs with high-grade features (necrosis, nuclear pleomorphism, high mitotic index, atypical mitogram) should be considered with more caution when considering prognosis [7]. At present, the prognosis of head and neck HCCC is clear, and as for pulmonary HCCC, which has similarities to head and neck, the prognosis of pulmonary HCCC still needs to be further investigated through the accumulated cases due to the limited number of cases of pulmonary HCCC. As of now, the prognosis for pulmonary HCCC is good.

However, it should be remembered that although the prognosis for pulmonary HCCC is good, metastases and recurrences can still be expected. A case of late recurrence of bronchial HCCC was reported by Wang et al. [14]. These findings suggest that bronchial HCCC risks late postoperative metastasis, even if it is an occult cancer, and therefore careful long-term follow-up should be advisable [5].

## Conclusion

Pulmonary HCCC is a low-grade malignant tumor. The majority of pulmonary HCCC occurs in middle-aged and elderly women over 40 years old. Clinical symptoms of pulmonary HCCC are non-specific, and some patients with pulmonary HCCC are even found accidentally during routine physical examinations. Pulmonary HCCC is most typically characterised by clear to eosinophilic tumour cells arranged in nests, ropes and trabeculae. IHC showed that all cases expressed epithelial markers, while myoepithelial markers were not expressed in all cases. *EWSR1::ATF1* is the most common gene fusion induced by t(12; 22)(q13; q12) mutations. Patients with pulmonary HCCC have a high survival rate, a good prognosis and recurrence is rare. Surgery is currently the main treatment for pulmonary HCCC, with other treatments including cryotherapy and laser therapy. The role of adjuvant chemotherapy and radiotherapy is unknown. Targeted therapies and immunotherapy should be explored in the future.

## Data Availability

All data generated or analyses during this study are included in this published article and its supplementary information files.
